# Dramatic response to alectinib in a patient with *ALK*‐rearranged squamous cell lung cancer

**DOI:** 10.1111/1759-7714.14092

**Published:** 2021-07-29

**Authors:** Jun Shiihara, Fumiyoshi Ohyanagi, Hikari Amari, Minemichi Toda, Hiroki Tahara, Motoi Yuzawa, Yuki Maeda, Motoko Nomura, Yoshiko Mizushina, Yoshiaki Nagai, Hiromitsu Ohta, Yasuhiro Yamaguchi

**Affiliations:** ^1^ Division of Respiratory Medicine, Clinical Department of Internal Medicine Jichi Medical University Saitama Medical Center Saitama Japan; ^2^ Division of Respiratory Medicine, Saitama Cancer Center Saitama Japan; ^3^ Division of Pulmonary Medicine, Department of Medicine Jichi Medical University Tochigi Japan

**Keywords:** alectinib, anaplastic lymphoma kinase, lung cancer, squamous cell cancer

## Abstract

Lung cancers with anaplastic lymphoma kinase (*ALK*) rearrangements are highly sensitive to treatment with ALK tyrosine kinase inhibitors (TKIs). Due to the very low rate of patients with squamous cell carcinoma enrolled in clinical trials, the efficacy of ALK inhibitors in patients with *ALK‐*rearranged squamous cell carcinoma in the lung remains unclear. Herein, we present the case of a 70‐year‐old female patient with squamous cell lung cancer harboring the echinoderm microtubule‐associated protein‐like 4 (*EML4*)‐*ALK* fusion gene. The patient was treated with the ALK‐TKI alectinib as first‐line regimen and achieved a dramatic response without severe adverse events, demonstrating alectinib as a therapeutic option for patients with ALK‐positive squamous cell carcinoma.

## Graphical abstract

The efficacy of ALK inhibitors in patients with *ALK‐*rearranged squamous cell carcinoma in the lung remains unclear. We present the case of a female patient with squamous cell lung cancer harboring the *EML4*‐*ALK* fusion gene. She was treated with the ALK‐TKI alectinib and achieved dramatic response without severe adverse events.

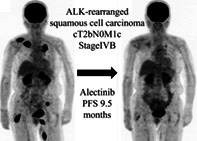

## INTRODUCTION

Specific molecular changes in certain lung cancers provide excellent opportunities for molecular targeted therapies. Echinoderm microtubule‐associated protein‐like 4 (*EML4*)‐anaplastic lymphoma kinase (*ALK*) rearrangement is found in 2%–7% of patients with non‐small cell lung cancer (NSCLC),^1^ a leading cause of cancer‐related deaths worldwide.^2^ However, *ALK* mutations are reported in only 0%–2.5% of patients with squamous cell cancer (SCC) of the lung.^3^

The ALK tyrosine kinase inhibitor (TKI) alectinib exhibits impressive single‐agent activity in ALK‐positive lung adenocarcinomas with an objective response rate (RR) of 70% and a median progression‐free survival of nearly 26 months.^4^ Due to the low percentage of patients with SCC enrolled in clinical trials, the efficacy of ALK TKIs in these patients is unclear. Herein, we present a patient with SCC harboring *EML4‐ALK* rearrangement successfully treated with alectinib.

## CASE REPORT

**FIGURE 1:**
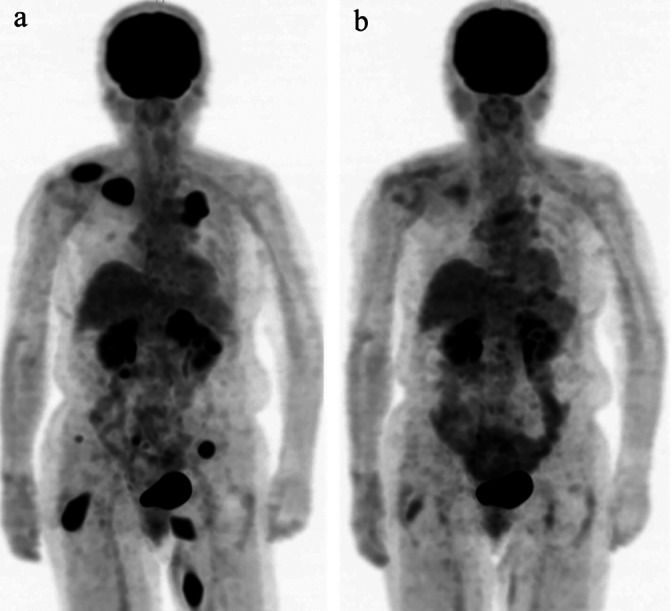
(a) Positron emission tomography‐computed tomography (PET‐CT) scan before the initiation of alectinib treatment showing a primary lesion in the left upper lung and multiple lung metastases. (b) PET‐CT scan obtained two months after the initiation of alectinib shows tumor shrinkage in the primary lesion and reduction in multiple metastatic sites

A 70‐year‐old female patient without a history of smoking presented with a subcutaneous tumor in the left thigh. Positron emission tomography (PET)‐computed tomography (CT) scans revealed a 4.0 × 4.2‐cm mass in the upper left lung lobe and multiple muscular and bone metastatic lesions (Figure 1(a)). She was diagnosed with stage IVB cancer (T2bN0M1c). Her performance status was 1 and serum carcinoembryonic antigen (CEA) level was 18.6 (normal range, 0–5.0) ng/ml.

**FIGURE 2:**
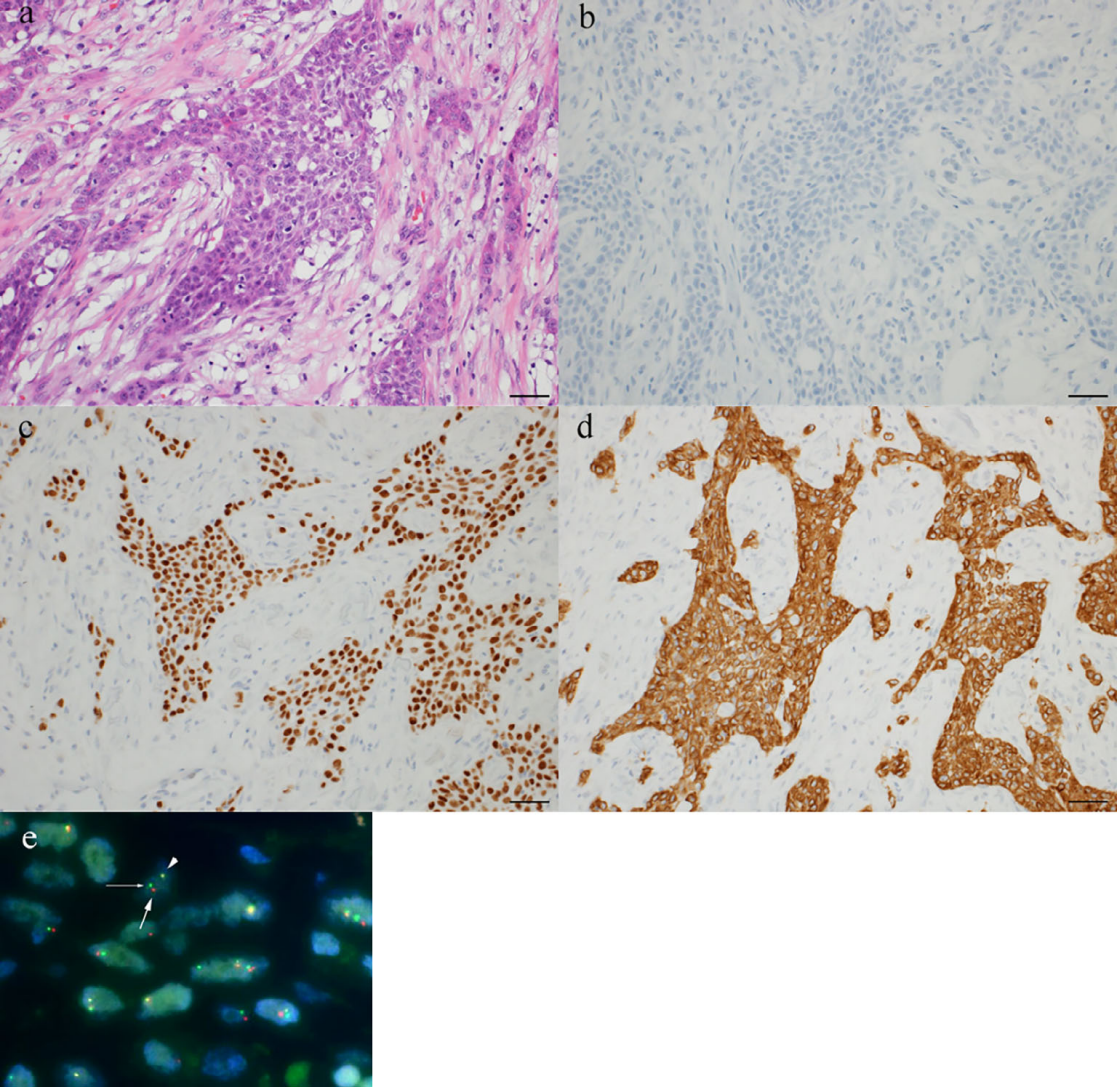
(a) Hematoxylin–eosin staining of squamous cell carcinoma in the subcutaneous tumor of left thigh. Scale bar, 50 μm. (b,c) Immunohistochemical studies show that the squamous component is (b) negative for thyroid transcription factor‐1 and (c) positive for p40. Scale bar, 50 μm. (d) Immunohistochemistry for anaplastic lymphoma kinase (ALK) using the D5F3 antibody shows positivity of the squamous cell carcinoma specimen. Scale bar, 50 μm. (e) Fluorescent in situ hybridization in the primary squamous cell carcinoma component using break‐apart probes for *ALK*. The 5′ green signal (narrow arrow) is breaking apart from the 3′ red signal (bold arrow), consistent with *ALK* rearrangement. The triangle indicates pseudo‐color signal by the *ALK* probe

Pathological examination of the resected subcutaneous tumor in the left thigh revealed moderately differentiated SCC with cornification (Figure 2(a)). Immunohistochemical analysis revealed diffuse and strongly positive staining for pan‐cytokeratin, cytokeratin 5/6, and p40, although the cells were negative for thyroid transcription factor‐1, CK7, napsin A, and CD56 (Figure 2(b),(c)). The tumor expressed wild‐type epidermal growth factor receptor (*EGFR*) based on the molecular analysis of exons 18–21 and was diffusely and strongly positive (3+) for ALK by intercalated antibody‐enhanced immunohistochemistry with an ALK detection kit (Nichirei Bioscience; Figure 2(d)). Based on fluorescence in situ hybridization (FISH) using break‐apart probes for *ALK* (Figure 2(e)), the patient was diagnosed with SCC harboring *EML4‐ALK* rearrangement and treated with alectinib (300 mg twice daily). Two months later, PET‐CT images revealed dramatic shrinkage in the size of primary tumor in the left upper lung lobe and reduction in the size of multiple muscular, bone, and skin metastases (Figure 1(b)); therefore, the patient was considered to have achieved partial response to alectinib according to the Response Evaluation Criteria in Solid Tumors guidelines (version 1.1). Although she experienced recurrence after a durable response of 9.5 months, there were no severe alectinib‐related adverse events.

## DISCUSSION

Lung SCC is the second most common histological NSCLC type, accounting for 20%–30% of all cases.^5^ In the last decade, advances in the identification of therapeutically relevant genetic alterations in *EGFR* ^6^ and *ALK* ^4^ have revealed that these events occur almost exclusively in adenocarcinoma, but not in SCC.^3^ Several recent case studies report *ALK* rearrangement in SCC^7^, ^8^, ^9^ based on conventional pathological diagnosis using small samples obtained by bronchoscopy or needle biopsy. According to the American Society of Clinical Oncology guidelines,^10^ molecular testing is recommended in fully resected lung cancer specimens because small biopsy specimens do not represent the precise characteristics of the entire tumor. The patient in this report was diagnosed based on the evaluation of fully resected tumor, with histological subtype confirmation by immunohistochemistry and ALK detection using immunohistochemistry and FISH. The patient in this report was a novel case of pure SCC without an adenocarcinoma component, with accompanying data on efficacy and duration of response to alectinib.

In Japan, the second‐generation ALK‐TKI alectinib was approved in July 2014 for patients with *ALK*‐rearranged NSCLC, based on the results of the AF‐001JP study.^11^ Furthermore, the results from two phase III trials, JALEX and ALEX, showed superior efficacy and lower toxicity of alectinib versus crizotinib for first‐line treatment of *ALK*‐positive NSCLC^4^, ^12^; the median progression‐free survival (PFS) and overall RR with alectinib were 25.7–34.1 months and 70%–80%, respectively, in these trials.

In the patient in this report, the CT scan three weeks after the initiation of alectinib revealed tumor shrinkage and the duration of response was 9.5 months. Compared with the previous *ALK*‐rearranged SCC case reports, the PFS of this case is longer than that of the four case reports treated with crizotinib (9, 7.1, 3, and 6 months, respectively)^7^, ^13^, ^14^, ^15^ and that of the nonresponse case treated with alectinib.^8^ It is also similar to that of the response case treated with alectinib (>11 months and > 9 months, respectively).^9^, ^16^ Our case and these case reports suggest that alectinib has a longer PFS, even in *ALK‐*rearranged SCC than crizotinib.

Previous studies have suggested that squamous cell carcinoma shows a poor response to TKIs than adenocarcinoma.^17^ A pooled analysis of EGFR‐TKI to *EGFR*‐mutated SCC revealed that the RR and median PFS were 30% and 3.1 months, respectively. These results are clearly inferior to the data for *EGFR*‐mutated adenocarcinoma, including the reported RR and median PFS of 70%–80% and 9–11 months, respectively.^6^ On the other hand, there is no large‐scale study that shows the efficacy of ALK‐TKI to *ALK*‐rearranged SCC because of the small number of patients. The PFS of this case was 9.5 months, which is shorter than the previously reported PFS of adenocarcinoma, but some SCC cases show promising PFS.^9^, ^16^ Further accumulation of clinical experience is needed to clarify the effectiveness of ALK‐TKI for *ALK*‐rearranged SCC. For the benefit of patients, genetic testing is important, even if the type of pathology is SCC.

The present case report has several limitations. The resected specimen for histological analysis was obtained from a subcutaneous tumor in the left thigh, and it remains possible that an adenocarcinoma component might have been present in other lesions, or that there may be histological discrepancies between the primary and metastatic lesions due to the heterogeneity and distribution of the tumor.

In conclusion, molecular testing for driver mutations should be considered in young patients with minimal or no smoking history, even if the histological findings correspond with those of SCC. Alectinib represents a reasonable treatment option in *ALK*‐rearranged lung SCC.

## CONFLICT OF INTEREST

None declared.
